# Impact of Multisystem Point-of-Care Ultrasonography in Guiding Clinical Decision Making and Interventions in an Indian Emergency Department: A Retrospective Study

**DOI:** 10.7759/cureus.109796

**Published:** 2026-05-28

**Authors:** Manali S Deshmukh, Siddharth Sahil, Shruti Menon, Sagar Sinha, Dhruva Reddy, Dattatray Bhusare, Kriti Gupta

**Affiliations:** 1 Emergency Medicine, Mahatma Gandhi Mission (MGM) Medical College and Hospital, Navi Mumbai, IND; 2 Traumatology and Surgery, Mahatma Gandhi Mission (MGM) Medical College and Hospital, Navi Mumbai, IND

**Keywords:** clinical decision-making, clinical impact, diagnostic accuracy, efast, emergency department(ed), india, lung ultrasound (lus), management outcomes, multisystem, pocus (point-of-care ultrasound)

## Abstract

Background

Point-of-care ultrasonography (POCUS) has evolved from bulky, stationary machines to compact handheld devices that can be used repeatedly without exposing patients to radiation. This makes it easier for clinicians to reassess patients over time and adjust diagnoses as clinical conditions change. It is now widely used in the emergency department (ED) as a quick bedside tool that supports immediate clinical evaluation, often avoiding the need to transfer patients to the radiology department. Its value is greatest in high-acuity situations, where timely decisions can be life-saving. Most of the available data, especially from India, focuses on individual applications rather than their broader, multisystem use in routine practice. Therefore, there is a need for research in that arena of POCUS. This study describes the association between multisystem POCUS findings and documented changes in diagnosis and management, and evaluates diagnostic accuracy against a composite clinical reference standard.

Materials and methods

This retrospective observational study was conducted in the ED of Mahatma Gandhi Mission (MGM) Medical College and Hospital, Navi Mumbai, an Indian tertiary healthcare center. The data used in this study were collected retrospectively from the hospital electronic medical records for all patients who underwent POCUS between January 2025 and June 2025. Different POCUS applications like extended focused assessment with sonography for trauma (eFAST), lung ultrasound (LUS), cardiac ultrasound, inferior vena cava assessment (IVC assessment), optic nerve sheath diameter (ONSD) measurement, transcranial Doppler (TCD), and airway ultrasound were included.

Results

Five hundred and seventy-three patients were screened, and 284 patients undergoing 390 POCUS examinations were included, among whom there was a change in diagnosis in 66 examinations (16.9%) and a change in management in 85 examinations (21.8%). Both outcomes changed simultaneously in 66 examinations (16.9%), management was refined without a diagnosis change in 19 examinations (4.9%), and the original plan was confirmed in 305 examinations (78.2%). ONSD measurement and TCD showed the highest rate of change in management (48.6% and 47.1%, respectively). IVC assessment had the highest accuracy (92.8%) and was the only application meeting both rule-in (positive likelihood ratio 12.54; 95% confidence interval 3.30-47.66) and rule-out (negative likelihood ratio 0.077; 95% confidence interval 0.030-0.200) thresholds. The TCD positive likelihood ratio (6.22; 95% confidence interval 0.96-40.23) was not statistically robust because the confidence interval crossed 1.0.

Conclusion

In this single-centre retrospective cohort, multisystem POCUS was associated with documented changes in diagnosis and management in a subset of emergency department examinations. Diagnostic performance varied across applications, with the most robust estimates observed for IVC assessment, while those for TCD and airway POCUS were limited by small sample sizes. Taken together, POCUS should be incorporated across EDs in India where it has the potential to improve patient care in a most practical and impactful way.

## Introduction

Point-of-care ultrasonography (POCUS) is a rapid, non-invasive bedside imaging modality that has become increasingly common in the emergency department (ED). It allows clinicians to evaluate a wide range of clinical conditions and make quicker, more accurate decisions, especially in critical situations [1].

POCUS is now an important part of everyday emergency medicine (EM) practice, helping clinicians to assess multiple organ systems in patients presenting to ED with conditions like undifferentiated shock, sepsis, trauma, dyspnoea, and chest pain, while also allowing quick bedside confirmation of endotracheal tube placement [2,3]. Its versatility makes it particularly valuable in time-sensitive and high-acuity scenarios.

The integration of POCUS into routine emergency care has been associated with enhanced timeliness of care, greater diagnostic accuracy, and reduced healthcare costs. It enables clinicians to address critical clinical questions and reduce the potential risks associated with advanced imaging modalities or invasive procedures [4].

Furthermore, a significant proportion of patients present in an unstable state, rendering transfer to the radiology suite challenging and potentially unsafe. In such circumstances, POCUS serves as an indispensable bedside diagnostic adjunct, allowing rapid evaluation without interrupting ongoing resuscitation, while reducing the risks associated with transporting unstable patients.

The American College of Emergency Physicians (ACEP) has recognised POCUS as a core competency for emergency physicians [4]. Despite its global adoption, comprehensive data from the Indian ED remains limited, with much of the available literature limited to trauma applications or single-organ studies [5,6]. This study was therefore undertaken to bridge this gap by evaluating the association between POCUS findings and documented changes in clinical decision-making and immediate management across a broad range of emergency presentations in a tertiary-care Indian ED. The applications studied include extended focused assessment with sonography for trauma (eFAST), lung ultrasound (LUS), cardiac ultrasound, inferior vena cava assessment (IVC assessment), optic nerve sheath diameter (ONSD) measurement, transcranial Doppler (TCD), and airway ultrasound. The primary objective was to describe the association between POCUS findings and documented changes in diagnosis and management, while the secondary objective was to assess its diagnostic accuracy against an appropriate composite reference standard.

## Materials and methods

Study design and setting

This retrospective observational study was conducted in the ED of Mahatma Gandhi Mission (MGM) Medical College and Hospital, Navi Mumbai, Maharashtra, a tertiary-level teaching hospital serving a predominantly urban population, between January 2025 and June 2025. The study was conducted in accordance with the principles of the Declaration of Helsinki, and Institutional Ethics Committee approval was obtained (Approval No. DHR-EC/2026/03/22, dated April 15, 2026).

Study population and patient selection

**Figure 1:**
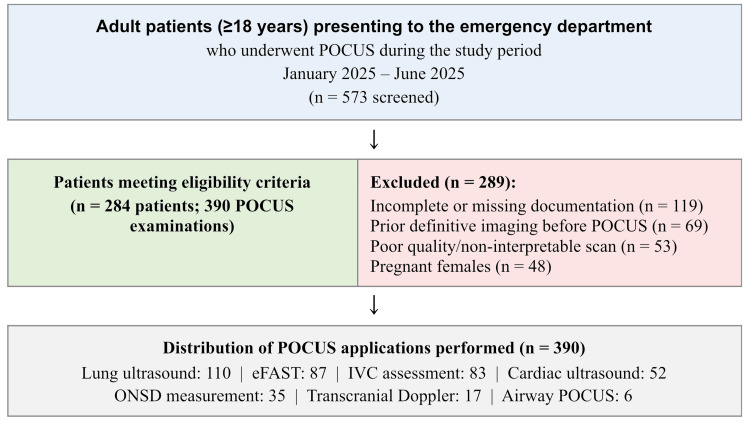
Patient selection flow diagram. POCUS: point-of-care ultrasonography, eFAST: extended focused assessment with sonography for trauma, IVC: inferior vena cava, ONSD: optic nerve sheath diameter.

All adult patients aged 18 years or older who presented to the ED and underwent POCUS as part of their clinical evaluation during the study period were identified from the hospital electronic medical records. A total of 573 patients were screened for eligibility. Patients with incomplete or missing clinical documentation, prior definitive imaging obtained before the POCUS evaluation, poor-quality or non-interpretable ultrasound examinations, and pregnant women were excluded from the study. After application of the inclusion and exclusion criteria, 284 patients undergoing a total of 390 POCUS examinations were included in the final analysis. The patient selection process is summarized in the patient selection flow diagram (Figure 1).

Sampling method

All adult patients who presented to the ED between the predefined study period and underwent POCUS as part of routine clinical care were sequentially screened in chronological order of presentation. No randomization, stratification, or selective enrolment was performed.

POCUS protocols and equipment

All POCUS examinations were performed by trained emergency medicine (EM) residents using the KOSMOS portable ultrasound system, Model HIUR2025004-07 (EchoNous, Redmond, WA, USA). Also, from EchoNous, the linear probe (KOSMOS LEXSA, 128 channel operating at 3-11 MHz with a center frequency of 7.5 MHz) was used for LUS and airway applications, while the phased-array probe (Torso-One operating at 1.5-4.5 MHz with a center frequency of 3 MHz) was used for IVC assessment, cardiac, and abdominal applications. For the purposes of this study, a trained resident was defined as one who had more than six months of ED POCUS experience and had attended a structured hands-on workshop together with supervised bedside teaching as part of routine departmental academic activity.

Standardized protocols were applied for each of the seven applications. eFAST involved assessment of pericardial, pleural, and peritoneal free fluid and pneumothorax using the subxiphoid, perihepatic, perisplenic, and pelvic windows together with bilateral anterior thoracic views. LUS consisted of a bilateral six-zone evaluation using the linear probe. Pulmonary edema was identified by the presence of diffuse bilateral B-lines (three or more per zone in multiple zones), pneumonia was identified as subpleural thickening with or without air bronchograms, pneumothorax was confirmed by identification of the lung point, and pleural effusion was identified as an anechoic pleural collection with the spine sign. Cardiac ultrasound included assessment of left ventricular systolic function, pericardial effusion, and right heart chamber dilatation using parasternal long axis, short axis, and apical four-chamber views. IVC assessment involved measurement of the maximum and minimum diameters of the IVC in the subcostal long-axis view during the respiratory cycle; a collapsibility index greater than 50% in spontaneously breathing patients, or a distensibility index less than 18% in mechanically ventilated patients, was considered indicative of volume responsiveness. ONSD measurement was performed bilaterally using the linear probe, 3 mm posterior to the posterior globe, and a threshold greater than 5 mm bilaterally was used to indicate raised intracranial pressure (ICP). TCD involved assessment of the middle cerebral artery mean flow velocity and pulsatility index through the temporal acoustic window, with a positive examination defined as a mean flow velocity greater than 120 cm/s, a pulsatility index greater than 1.2, or absent or reversed diastolic flow. Airway POCUS involved the identification of esophageal intubation using a linear probe placed over the anterior neck. A positive examination was defined by the presence of the bilateral double-tract sign, whereas a negative examination was defined by the identification of a single air-mucosa interface with comet-tail artifact, indicating tracheal placement.

For the purpose of diagnostic accuracy analysis, each POCUS application was classified as positive or negative based on the predefined sonographic criteria described above. A positive examination was defined by the presence of the specified abnormal findings for that application, while a negative examination indicated their absence. These binary classifications were then compared against the final clinical composite diagnosis to derive true positive, false positive, true negative, and false negative counts for each application.

Reference standard

All POCUS examination findings were compared with a final clinical composite diagnosis, which was considered the reference standard. This composite reference standard was determined by integrating all available clinical and investigational information obtained during the ED visit and subsequent inpatient stay. The findings contributing to the composite reference standard include: standard imaging modalities (computed tomography, magnetic resonance imaging, chest radiograph), clinical examination, intra-operative surgical findings (for unstable patients who proceeded to operating room), formal ultrasonography, formal transthoracic echocardiography, relevant laboratory investigations, clinical hemodynamic response to fluid challenge or vasopressor initiation, supported by invasive monitoring, and waveform capnography combined with direct laryngoscopy (for airway POCUS). Final clinical diagnoses used to derive the composite reference standard were based on documented assessments by the attending EM physicians. As POCUS findings were part of the medical record during clinical review, formal blinding to POCUS results was not feasible.

Data collection

Patient records were reviewed from the hospital electronic health record system. All patient data were de-identified prior to analysis to ensure confidentiality. For each patient, the following variables were recorded: age, sex, presenting complaint, POCUS application performed, pre-POCUS working diagnosis, pre-POCUS management plan, POCUS finding with positive or negative classification, post-POCUS revised diagnosis, post-POCUS management plan, management change classification, type of change, specific intervention performed or avoided, and the reference standard result.

Primary and secondary outcomes

The study had two co-primary outcomes that were evaluated independently. The first was a change in diagnosis, defined as a difference between the pre-POCUS working diagnosis and the post-POCUS revised diagnosis at the level of disease label; confirmation of a suspected diagnosis was not counted as a change. The second was a change in management, defined as the proportion of examinations in which the documented post-POCUS clinical action differed from the documented pre-POCUS plan. Management outcome was categorised as: yes- management changed (POCUS led to a different clinical action); confirmed original plan (POCUS confirmed the pre-existing plan was appropriate); or no change. The type of management change was further classified as therapeutic (medication started, changed, or stopped), diagnostic (imaging obtained or avoided), procedural (procedure performed or avoided), resuscitative (fluid strategy or vasopressor modified), or dispositional (admission level changed- ward or Intensive care unit admission). Where a patient's management change was observed in more than one category simultaneously, it was recorded as multiple management categories. The two primary outcomes yielded four possible cross-tabulation categories: both diagnosis and management changed, diagnosis unchanged but management changed, diagnosis changed but management unchanged, and neither diagnosis nor management changed.

The secondary outcome was the diagnostic accuracy of POCUS compared with the final clinical composite diagnosis for each application, expressed as sensitivity, specificity, positive predictive value, negative predictive value, overall accuracy, positive likelihood ratio, and negative likelihood ratio, each presented with 95% confidence intervals.

Statistical analysis

Categorical data are expressed as numbers and percentages. The unit of analysis was the individual POCUS examination, with one application per row, where a patient underwent more than one application; each was treated as an independent observation, since each addressed a distinct clinical question with an independent reference comparison. For the co-primary outcomes, the proportion of patients with a change in diagnosis and a change in management was calculated overall and per application, with cross-tabulation presented as a four-quadrant analysis. For the secondary outcome, sensitivity, specificity, positive predictive value, negative predictive value, overall accuracy, positive likelihood ratio, and negative likelihood ratio were calculated for each application separately with 95% confidence intervals. Diagnostic accuracy statistics were not pooled across applications, as each application addressed a different clinical question and pooling would be methodologically inappropriate. Wilson score intervals were used for proportions, while the log-transformation method was used for likelihood ratios. A positive likelihood ratio greater than ten was considered a clinically useful rule-in test, while a negative likelihood ratio less than 0.1 was considered a clinically useful rule-out test. Where the 95% confidence interval of a likelihood ratio crossed 1.0, the result was considered not statistically robust and interpreted with caution. Statistical analysis was performed using MedCalc software version 20.217 (MedCalc Software Ltd, Ostend, Belgium).

## Results

Patient characteristics

Out of 573 patients screened, 284 met the inclusion criteria and were included in the final analysis, contributing 390 POCUS examinations. The study population comprised 176 men (62.0%) and 108 women (38.0%), with a median age of 42 years (range 18 to 75). Acute respiratory distress was the most common ED presentation, accounting for 110 examinations (28.2%) performed as LUS. Trauma was the second most common presentation, with 87 examinations (22.3%) performed as eFAST. Other presentations included hemodynamic instability and sepsis (83 examinations, 21.3%; IVC assessment), acute coronary syndrome and other cardiac symptoms (52 examinations, 13.3%; cardiac ultrasound), acute neurological dysfunction with suspected raised ICP (35 examinations, 9.0%; ONSD measurement), cerebrovascular emergencies (17 examinations, 4.4%; TCD), and post-intubation airway confirmation (six examinations, 1.5%; airway POCUS). Patient characteristics are summarized in Table 1.

**Table 1:** General characteristics of the included study population. IQR: interquartile range, POCUS: point-of-care ultrasonography.

Characteristic	Value	Notes
Total patients screened	573	—
Total patients included	284	Met the inclusion and exclusion criteria
Male, n (%)	176 (62.0%)	—
Female, n (%)	108 (38.0%)	—
Median age, years	42	Range 18 to 75; IQR approximately 28 to 58
Total POCUS scans	390	Across seven clinical applications

Primary outcomes: change in diagnosis and change in management

Across 390 POCUS scans, we observed a change in diagnosis in 66 scans (16.9%) and a change in management in 85 scans (21.8%). The cross-tabulation of these co-primary outcomes is presented in Table 2 and reveals three distinct clinical patterns. In 66 scans (16.9%), both the diagnosis and management changed. A positive POCUS finding revealed previously unsuspected pathology, or a negative finding excluded the initially suspected diagnosis that resulted in both a change in diagnosis and a corresponding new clinical action. In 19 scans (4.9%), the diagnosis label remained the same, but the management was modified, reflecting severity-guided refinement (for instance, in a patient with acute neurological dysfunction, a normal ONSD assessment reduced suspicion of markedly raised ICP, supported proceeding with lumbar puncture in the appropriate clinical context, and helped avoid unnecessary empirical osmotherapy without altering the underlying diagnostic label). In the remaining 305 scans (78.2%), neither the diagnosis nor the management changed, and the original clinical plan was confirmed as appropriate. Notably, no scan produced a change in diagnosis without a corresponding change in management, an observation discussed in detail below. 

**Table 2:** Change in diagnosis and management by application (co-primary outcomes). Diagnosis changed: Post-POCUS diagnosis differed from the initial (pre-POCUS) diagnosis. Management changed: Post-POCUS management plan differed from the initial (pre-POCUS) plan. Both changed: Both diagnosis and management changed after POCUS. Original plan confirmed: Both diagnosis and management remained unchanged after POCUS. Diagnosis changed, management unchanged: Diagnosis changed after POCUS, but the management plan remained the same. eFAST: extended focused assessment with sonography for trauma, LUS: lung ultrasound, IVC: inferior vena cava, ONSD: optic sheath nerve diameter, TCD: transcranial Doppler, POCUS: point-of-care ultrasonography.

Application	N	Diagnosis changed, n (%)	Management changed, n (%)	Both changed, n (%)	Original plan confirmed, n (%)	Diagnosis changed, management unchanged, n (%)
eFAST	87	15 (17.2%)	16 (18.4%)	15 (17.2%)	71 (81.6%)	0 (0.0%)
LUS	110	22 (20.0%)	22 (20.0%)	22 (20.0%)	88 (80.0%)	0 (0.0%)
Cardiac ultrasound	52	12 (23.1%)	12 (23.1%)	12 (23.1%)	40 (76.9%)	0 (0.0%)
IVC assessment	83	9 (10.8%)	9 (10.8%)	9 (10.8%)	74 (89.2%)	0 (0.0%)
ONSD measurement	35	3 (8.6%)	17 (48.6%)	3 (8.6%)	18 (51.4%)	0 (0.0%)
TCD	17	4 (23.5%)	8 (47.1%)	4 (23.5%)	9 (52.9%)	0 (0.0%)
Airway POCUS	6	1 (16.7%)	1 (16.7%)	1 (16.7%)	5 (83.3%)	0 (0.0%)
Overall	390	66 (16.9%)	85 (21.8%)	66 (16.9%)	305 (78.2%)	0 (0.0%)

ONSD measurement and TCD showed the highest management change rates (48.6% and 47.1%, respectively). In both, much of the impact arose from severity refinement rather than diagnostic reclassification. Most patients remained labelled as having acute neurological dysfunction, but negative POCUS findings supported a more targeted workup. Cardiac ultrasound resulted in a change in management of 12 out of 52 examinations (23.1%), principally through the identification of unsuspected pericardial effusion or by reducing suspicion for acute coronary syndrome when cardiac POCUS findings were unremarkable and the overall clinical assessment favored alternative non-cardiac causes of chest pain. LUS resulted in management change in 22 of 110 examinations (20.0%), predominantly by avoiding empirical pharmacotherapy when POCUS excluded the initially suspected diagnosis. eFAST changed management in 16 of 87 trauma examinations (18.4%) by detecting unexpected hemoperitoneum, pneumothorax, or hemopericardium that were not initially suspected. IVC assessment changed management in nine of 83 scans (10.8%), mainly by redefining the hemodynamic status. Among six post-intubation airway POCUS examinations, five had esophageal intubation confirmed by the composite reference standard (capnography combined with direct laryngoscopy). Airway POCUS correctly identified four of these five cases by demonstrating the double-tract sign (one false negative), while the single patient without esophageal intubation was correctly identified by the presence of a single air-mucosa interface with comet-tail artifact (no false positives). All five patients with confirmed esophageal intubation underwent immediate reintubation.

The categorization of management changes is presented in Table 3. The single most frequent change was avoidance of diagnostic imaging, followed by therapeutic medication change and procedural intervention. Seven patients had simultaneous changes across more than one category and were grouped under multiple management categories, for example, when POCUS triggered both an emergency procedure and a concurrent change in resuscitation strategy.

**Table 3:** Types of management changes observed among the 85 examinations that resulted in a change in management. The category 'multiple management categories' denotes scans in which point-of-care ultrasonography triggered more than one simultaneous type of clinical action change (for example, a procedure was performed, a medication was started, and a disposition decision was made simultaneously). CT: computed tomography, HRCT: high resolution computed tomography, POCUS: point-of-care ultrasonography, eFAST: extended focused assessment with sonography for trauma, MTP: massive transfusion protocol, LUS: lung ultrasound.

Type of management change	n	%	Examples
Diagnostic — imaging avoided	26	30.6%	HRCT thorax, CT pulmonary angiography avoided.
Therapeutic — medication changed	19	22.4%	Drug class switched (for example, diuretics changed to bronchodilators; aggressive fluid resuscitation changed to inotrope therapy).
Procedural — procedure performed	13	15.3%	Laparotomy, tube thoracostomy, pericardiocentesis or thoracocentesis performed.
Multiple management categories	7	8.2%	More than one type of clinical action change occurred simultaneously.
Resuscitation — fluid strategy changed	6	7.1%	Aggressive fluids are initiated or withheld based on the inferior vena cava phenotype.
Diagnostic — imaging escalated	4	4.7%	Urgent CT or CT pulmonary angiography triggered by an unexpected POCUS finding.
Procedural — procedure avoided	3	3.5%	Thoracocentesis or tube thoracostomy is avoided after POCUS exclusion of effusion or pneumothorax.
Therapeutic — medication started	3	3.5%	New drug class initiated (osmotherapy, vasopressor, or anticoagulation).
Disposition — intensive care admission	2	2.4%	Intensive care admission for traumatic hemopericardium for monitoring.
Disposition — admit to ward	1	1.2%	Suspected abdominal trauma with initial hemodynamic instability; negative eFAST and clinical stabilisation prompted de-escalation to ward care.
Therapeutic — medication stopped	1	1.2%	MTP for presumed hemorrhagic shock stopped after evidence of B-lines on LUS indicating fluid overload.
Total	85	100%	—

Secondary outcome: diagnostic accuracy

Diagnostic accuracy was assessed against the final clinical composite diagnosis for each application separately and is reported in Table 4. In examinations classified as false negatives, the trigger for further surgical or medical management was ongoing clinical deterioration, persistent or evolving symptoms, or abnormalities on subsequent investigations such as CT, formal echocardiography, or serial troponins, rather than the POCUS finding itself. eFAST demonstrated high sensitivity and a low negative likelihood ratio, supporting its use as a reliable rule-out tool for clinically significant hemoperitoneum, hemothorax, hemopericardium, and pneumothorax in trauma. The highest positive likelihood ratio was seen in LUS, although the wide confidence interval reflects the small number of true negative observations. Cardiac ultrasound demonstrated high sensitivity and a clinically useful negative likelihood ratio. IVC assessment was the only application to satisfy both the rule-in (positive likelihood ratio greater than ten) and rule-out (negative likelihood ratio less than 0.1) thresholds simultaneously. ONSD measurement showed good sensitivity for raised ICP. TCD showed a positive likelihood ratio of 6.22, suggesting potential diagnostic value. However, the 95% confidence interval (0.96-40.23) is wide and crosses 1.0, meaning the result is imprecise and not statistically robust. Given the small sample size (n = 17), this finding should be interpreted with caution and does not provide reliable evidence of diagnostic utility at the population level. Airway POCUS achieved 100% specificity and 100% positive predictive value for esophageal intubation; the positive likelihood ratio could not be calculated conventionally because there were no false positive examinations.

**Table 4:** Diagnostic accuracy of point-of-care ultrasonography by application with 95% confidence intervals (secondary outcome). Each application was compared independently against the final clinical composite diagnosis. *Transcranial Doppler positive likelihood ratio confidence interval (0.96–40.23) crosses 1.0, indicating that this result is not statistically robust and should be interpreted with caution. Airway point-of-care ultrasonography positive likelihood ratio is not calculable conventionally because there were no false positive examinations. eFAST: extended focused assessment with sonography for trauma, LUS: lung ultrasound, IVC: inferior vena cava, ONSD: optic nerve sheath diameter, TCD: transcranial Doppler, POCUS: point-of-care ultrasonography. CI: confidence interval, +LR: positive likelihood ratio, −LR: negative likelihood ratio, NPV: negative predictive value, PPV: positive predictive value.

Application	N	Prevalence	Sensitivity % (95% CI)	Specificity % (95% CI)	Accuracy %	PPV %	NPV %	+LR (95% CI)	−LR (95% CI)
eFAST	87	67.8%	95.7% (88.0–98.5)	77.8% (54.8–91.0)	92.0%	94.3%	82.4%	4.30 (1.81–10.23)	0.056 (0.018–0.174)
LUS	110	72.7%	80.0% (70.0–87.3)	96.7% (83.3–99.4)	84.5%	98.5%	64.4%	24.00 (3.48–165.4)	0.207 (0.133–0.322)
Cardiac ultrasound	52	55.8%	95.2% (84.2–98.7)	70.0% (39.7–89.2)	90.4%	93.0%	77.8%	3.17 (1.23–8.20)	0.068 (0.017–0.279)
IVC assessment	83	67.5%	92.9% (83.0–97.2)	92.6% (76.6–97.9)	92.8%	96.3%	86.2%	12.54 (3.30–47.66)	0.077 (0.030–0.200)
ONSD measurement	35	65.7%	91.3% (73.2–97.6)	83.3% (55.2–95.3)	88.6%	91.3%	83.3%	5.48 (1.54–19.54)	0.104 (0.027–0.402)
TCD	17	52.9%	77.8% (45.3–93.7)	87.5% (52.9–97.8)	82.4%	87.5%	77.8%	6.22 (0.96–40.23)*	0.254 (0.073–0.886)
Airway POCUS	6	83.3%	80.0% (37.6–96.4)	100% (20.7–100.0)	83.3%	100%	50.0%	Not calculable	0.200

## Discussion

This retrospective study examined the clinical impact and diagnostic accuracy of multisystem POCUS across seven applications in a tertiary care urban ED in India. POCUS findings were compared against a final clinical composite diagnosis incorporating all available imaging, laboratory, surgical, and clinical data. The study found that POCUS resulted in a change in diagnosis in 16.9% and a change in management in 21.8% of presentations, with management refinement without a diagnosis change in a further 4.9%. The original clinical plan was confirmed in 78.2% of patients. Despite being a retrospective single-center study, our findings suggest that POCUS has an important role in the emergency department.

An important methodological observation is that the “diagnosis changed, management unchanged” category was not observed in any of the 390 POCUS scans and requires careful interpretation. We classified management changes into 11 groups, including therapeutic, diagnostic, procedural, resuscitative, and disposition-related actions, with an additional category for cases involving more than one type of change. Given the breadth of this taxonomy, any clinically meaningful change in diagnosis was almost invariably accompanied by at least one recordable change in clinical action. In routine emergency practice, this is an expected pattern as a clinically significant diagnosis change without any modification of management would be unusual and arguably clinically incomplete. The absence of this category, therefore, reflects how POCUS findings are a valuable modality in immediate decision-making, rather than being a methodological artefact. Future studies using more granular management outcome definitions may be able to identify subtle scenarios in which a diagnosis change occurs without an immediate management change.

The highest management change rates were observed in the neurological applications. For ONSD measurement, most management changes (14 of 17) occurred without a change in diagnosis label, illustrating how POCUS impact can occur through severity refinement, for example, by allowing safe lumbar puncture and avoiding empirical osmotherapy. For TCD, although the 47.1% change in management was substantial, the diagnostic accuracy is less reliable because of the small sample (n=17) and a wide confidence interval for the positive likelihood ratio that includes 1.0. The observed management change rate likely reflects the role of TCD as a bedside screening and monitoring modality that triggers further investigation, rather than being a standalone diagnostic test. This interpretation aligns with previous studies describing TCD as a screening tool that needs confirmation with definitive neuroimaging [7].

In our study, eFAST resulted in a change in management in 18.4% of patients, exceeding the ~10% therapeutic impact reported in earlier trauma center studies [8]. This discrepancy between the studies likely reflects differences in patient selection, operator expertise, and how “change in management” is defined. The higher rate we reached was primarily driven by the identification of unexpected pathology in patients whose initial clinical assessment suggested injury confined to a single anatomical region. For example, free intraperitoneal fluid or pneumothorax in patients initially thought to have isolated head or limb injuries frequently prompted escalation of care and major interventions. Together, these results show that eFAST is better viewed as a whole-body trauma screening tool, rather than being restricted to abdominal assessment. An additional pragmatic advantage observed in our cohort was time efficiency. In selected unstable trauma patients, a positive eFAST finding allowed direct transfer to the operating theatre without waiting for CT, which can be a rate-limiting step in busy tertiary EDs.

IVC assessment demonstrated favorable diagnostic characteristics, with both positive and negative likelihood ratios supporting its utility in ruling in and ruling out fluid responsiveness, consistent with prior literature [9].

Previous studies on LUS have shown a significant impact on decision-making and therapeutic management, which aligns with our study, where LUS led to a 20% change in management, mainly because it helped revise the diagnosis [10]. Meta-analyses done previously have demonstrated high diagnostic accuracy with LUS reporting, elevated positive likelihood ratios, underscoring its effectiveness as a rule-in modality, especially in differentiating pulmonary edema and pneumonia from alternate diagnoses, including acute respiratory presentations [11,12]. The high positive likelihood ratio of LUS (24.00) reflects strong rule-in capability when the examination is positive, although the wide confidence interval is consistent with the limited number of true negative observations.

Cardiac ultrasound resulted in management change in 23.1% of ultrasound examinations, primarily through the identification of unsuspected pericardial effusion and reclassification of patients with suspected acute coronary syndrome to alternative diagnoses such as acute gastritis or non-ischemic chest pain. The clinical importance of these reclassifications lies in their direct influence on antiplatelet, anticoagulation, and admission decisions, as highlighted in prior literature [13]. The cardiac ultrasound positive likelihood ratio of 3.17 (95% confidence interval 1.23-8.20) does not meet the conventional rule-in threshold, but the negative likelihood ratio of 0.068 confirms its strong rule-out capability for major cardiac pathology.

Airway POCUS demonstrated high specificity and correctly identified four of five esophageal intubations, facilitating prompt reintubation in all confirmed cases, although one esophageal intubation was missed on POCUS assessment. This likely reflects the high pre-existing clinical suspicion in most patients and the small sample size. This is consistent with prior evidence, including the systematic review by Chou et al., which demonstrated the high diagnostic value of ultrasonography for confirming endotracheal tube placement [14].

Recent randomised ED evidence comparing trans-tracheal ultrasound, lung-sliding ultrasound, and diaphragm ultrasound for post-intubation confirmation has shown high diagnostic performance across all three techniques, with trans-tracheal ultrasound providing the shortest confirmation time. Although the present airway POCUS subgroup was small, its findings are consistent with the growing evidence supporting airway ultrasound as a rapid adjunct for detecting esophageal intubation and supporting early post-intubation verification [15].

The findings of these prior studies are consistent with our results, but differences in case-mix, training context, and reference standards should be accounted for as well.

Smith et al. similarly described the expanding role of bedside ultrasound in Indian ED, while highlighting the structural barriers that continue to limit its widespread adoption [5]. A wider contextual issue is the absence of a national standardised POCUS training curriculum in India. EM residents are increasingly expected to perform POCUS in clinical practice without a defined national competency framework or minimum supervised scan requirement. The present study used an internal departmental definition of training (more than six months of emergency department POCUS experience, hands-on workshop, supervised bedside teaching), but this cannot substitute for a validated national standard. Operator-dependent variability is therefore an acknowledged source of potential bias. The development and implementation of a uniform national POCUS competency framework should be a training priority [5,6,16].

Strengths and limitations

The strengths of our study are its multi-application scope across seven distinct POCUS domains, use of a clinically grounded composite reference standard rather than a single per modality standard, transparent and independent reporting of both co-primary outcomes with cross-tabulation established by attending emergency physician, the use of consecutive sampling to minimise selection bias, reporting of 95% confidence intervals across all accuracy metrics and the real world applicability of the findings to the Indian tertiary ED setting.

Limitations of our study include its retrospective nature, single-center study design, selection bias due to the exclusion of patients with incomplete documentation or prior imaging, and the application of multiple investigations with varying levels of accuracy across various POCUS modalities, which raises the possibility of differential verification and partial verification bias, smaller sample sizes for TCD and airway POCUS make the result statistically unreliable and warrant a larger study. In-hospital mortality data were not collected, limiting the assessment of outcomes, and blinding between POCUS findings and the final clinical diagnosis was not feasible, introducing potential incorporation bias. The cohort included only patients in whom POCUS was performed at the treating clinician’s discretion, rather than all ED patients with the relevant clinical presentations. This reflects real-world POCUS use but introduces clinician-driven selection bias. Additionally, given the retrospective design, our analysis reflects associations between POCUS findings and documented changes in diagnosis or management, and a direct causal relationship cannot be established. Finally, the lack of a national standardised POCUS training framework in India may lead to operator-dependent variability even within a single department.

## Conclusions

In our setting, multisystem POCUS performed by trained EM residents seemed to play a pivotal role in clinical decision-making in the ED. It not only contributed to changes in diagnosis but also influenced management decisions, while confirming the initial management plan in most patients. Overall, it demonstrated strong diagnostic performance across most applications, particularly as a reliable rule-out tool, with select modalities also showing robust rule-in capability. 

The findings of our study highlight the need to integrate POCUS into the healthcare system to support its consistent, effective use in routine emergency care, especially in resource-limited settings such as India. However, larger prospective multi-centre studies are needed to confirm and extend these findings, and to establish their generalizability to other settings within India and beyond.
